# Development and Validation of Deep Learning–Based Infectivity Prediction in Pulmonary Tuberculosis Through Chest Radiography: Retrospective Study

**DOI:** 10.2196/58413

**Published:** 2024-11-07

**Authors:** Wou young Chung, Jinsik Yoon, Dukyong Yoon, Songsoo Kim, Yujeong Kim, Ji Eun Park, Young Ae Kang

**Affiliations:** 1 Department of Pulmonary and Critical Care Medicine Ajou University School of Medicine Suwon Republic of Korea; 2 Department of Integrative Medicine Yonsei University College of Medicine Seoul Republic of Korea; 3 Department of Biomedical Systems Informatics Yonsei University College of Medicine Seoul Republic of Korea; 4 Institute for Innovation in Digital Healthcare Severance Hospital Seoul Republic of Korea; 5 Center for Digital Health Yongin Severance Hospital Yonsei University Health System Yongin Republic of Korea; 6 Department of Internal Medicine Yonsei University College of Medicine Seoul Republic of Korea

**Keywords:** pulmonary tuberculosis, chest radiography, artificial intelligence, tuberculosis, TB, smear, smear test, culture test, diagnosis, treatment, deep learning, CXR, PTB, management, cost effective, asymptomatic infection, diagnostic tools, infectivity, AI tool, cohort

## Abstract

**Background:**

Pulmonary tuberculosis (PTB) poses a global health challenge owing to the time-intensive nature of traditional diagnostic tests such as smear and culture tests, which can require hours to weeks to yield results.

**Objective:**

This study aimed to use artificial intelligence (AI)–based chest radiography (CXR) to evaluate the infectivity of patients with PTB more quickly and accurately compared with traditional methods such as smear and culture tests.

**Methods:**

We used DenseNet121 and visualization techniques such as gradient-weighted class activation mapping and local interpretable model-agnostic explanations to demonstrate the decision-making process of the model. We analyzed 36,142 CXR images of 4492 patients with PTB obtained from Severance Hospital, focusing specifically on the lung region through segmentation and cropping with TransUNet. We used data from 2004 to 2020 to train the model, data from 2021 for testing, and data from 2022 to 2023 for internal validation. In addition, we used 1978 CXR images of 299 patients with PTB obtained from Yongin Severance Hospital for external validation.

**Results:**

In the internal validation, the model achieved an accuracy of 73.27%, an area under the receiver operating characteristic curve of 0.79, and an area under the precision-recall curve of 0.77. In the external validation, it exhibited an accuracy of 70.29%, an area under the receiver operating characteristic curve of 0.77, and an area under the precision-recall curve of 0.8. In addition, gradient-weighted class activation mapping and local interpretable model-agnostic explanations provided insights into the decision-making process of the AI model.

**Conclusions:**

This proposed AI tool offers a rapid and accurate alternative for evaluating PTB infectivity through CXR, with significant implications for enhancing screening efficiency by evaluating infectivity before sputum test results in clinical settings, compared with traditional smear and culture tests.

## Introduction

Pulmonary tuberculosis (PTB) is a global health concern that continues to challenge public health systems. In 2022, it affected approximately 10.6 million people, with an incidence rate of 133 per 100,000 people. Notably, it has claimed the lives of approximately 1.13 million patients who are HIV negative and 167,000 patients who are HIV positive, underscoring its high mortality rate [1].

Many people are infected with the tuberculosis bacteria, but not all develop active tuberculosis. Identifying this asymptomatic infection is a major challenge in public health [2]. Traditional tuberculosis diagnostic methods include smear and culture tests, which may be time-consuming or exhibit low sensitivities, resulting in missed early diagnosis. As tuberculosis bacteria are airborne, quickly identifying and isolating patients with active tuberculosis is crucial for preventing further spread. In addition, rapid diagnosis allows for the timely and appropriate treatment of patients, increasing the success rate and minimizing complications [3].

The sensitivity of traditionally used smear tests is highly variable, ranging from 50% to 60% [4]. Thus, these tests may not detect PTB, particularly when there are low bacterial loads, and are unreliable for early diagnosis. This problem can increase the risk of missing PTB in its early stages and result in patients with asymptomatic or mild symptoms not receiving appropriate diagnosis and treatment [5].

Although culture tests exhibit a high sensitivity of approximately 80% [6], they require several weeks and sophisticated laboratory facilities, which may not be readily available, especially in resource-limited settings. However, these delays can hinder the proper initiation of treatment for people with active PTB, which can eventually lead to poorer health and increased mortality. When a rapid and accurate diagnosis is not made, PTB can easily spread to others, imposing a significant burden on public health and complicating control and management efforts [7]. Furthermore, traditional sputum testing has exhibited a sensitivity of 52% for patients undergoing treatment [8].

Computed tomography (CT) is an important diagnostic tool that provides detailed information of lung structures for lung disease evaluations, particularly PTB [9]. However, despite its diagnostic use, using CT for PTB diagnosis has several critical issues, including limited availability, high costs, and radiation exposure concerns [10]. For example, a lack of imaging facilities necessary for CT scans may exist in resource-limited countries. This limitation restricts access to crucial diagnostic imaging, delaying the diagnosis and treatment of patients in these regions. Consequently, health crises may be exacerbated by allowing diseases to spread more widely without timely intervention. Recent advancements in medical imaging, specifically chest radiography (CXR), have contributed significantly to the early detection and management of PTB. The use of CXR as a primary PTB screening tool has been reaffirmed by its high sensitivity despite its specificity limitations. Various studies have demonstrated that CXR is instrumental for identifying PTB, especially in vulnerable populations that exhibit a notably higher disease prevalence than the general population [11,12].

The use of CXR for assessing PTB infectivity offers several key advantages over CT as x-ray equipment is more widely available and simple to operate. In addition, it enables swift diagnosis and timely treatments, which is particularly advantageous for regions with limited access to advanced medical equipment. Moreover, CXR involves significantly less radiation exposure than CT, which is crucial for sensitive groups such as pregnant women and children, who are more susceptible to the adverse effects of radiation. CXR is also highly suitable for large-scale screening. Thus, its rapid processing and cost efficiency make it ideal for mass PTB screening initiatives that are vital for curbing the spread of tuberculosis.

The advent of deep learning technologies has opened new avenues for enhancing the diagnosis and management of patients with PTB [13]. Our study leverages these advancements to differentiate infectious and noninfectious PTB cases using CXR, which is critical for determining appropriate quarantine measures and controlling the spread of tuberculosis [14]. Conventional PTB detection approaches are primarily used to identify the presence or absence of the disease. However, the infectious status of PTB plays a crucial role in public health, particularly for making quarantine decisions and preventing its spread [15].

This study was motivated by the need for diagnostic tools that can provide insights into the infectivity of PTB cases beyond mere detection. The aim of our artificial intelligence (AI) tool is to bridge the gap in PTB management in resource-limited countries by using CXR to evaluate the infectivity of patients with PTB more quickly and accurately than when using smear and culture tests. The proposed tool can significantly impact public health strategies and offer a more targeted approach for quarantine decisions.

## Methods

### Patients and Infectivity Labeling

The cohort comprised patients who met the following criteria: (1) first PTB diagnosis between January 2004 and June 2023, (2) underwent sputum examinations, (3) CXR performed within –60 to +180 days from the initial diagnosis date, (4) at least 1 positive sputum sample (smear or culture), and (5) received a successful 6-month treatment course (no treatment after the seventh month).

For positive labels, infectious CXR images were defined as those obtained from the initial diagnosis date to the last positive sputum test result. For negative labels, we used CXR images obtained after the conversion date, which was the day when the sputum test result changed from positive to negative. Specifically, a 1-month gray zone was established following the next sputum test date after the conversion date, that is, the CXR image obtained after the second negative sputum test result post conversion was classified as noninfectious.

**Figure 1:**
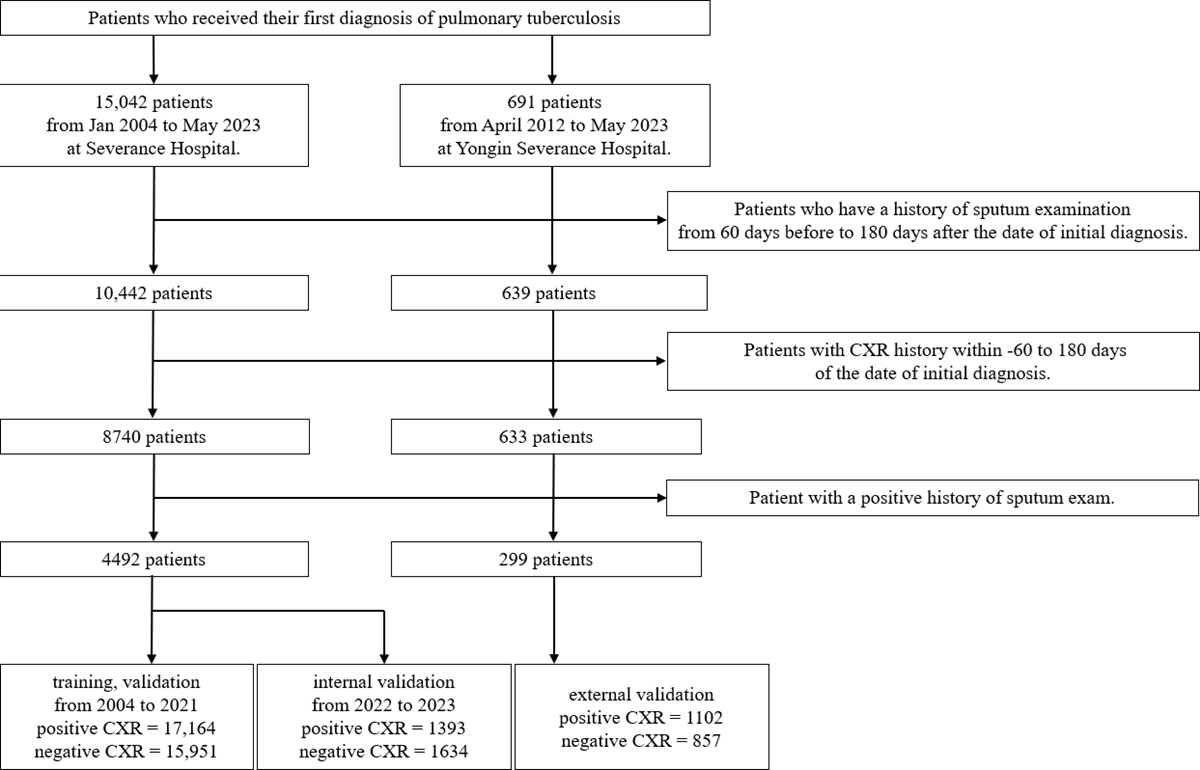
Systematic flowchart of the patient selection criteria and process used in this study. CXR: chest radiography.

We used data from Severance Hospital for the training dataset. Internal validation was performed using temporal validation, and external validation was conducted using data from Yongin Severance Hospital, which has different geographical and scale characteristics compared with Severance Hospital. The data of patients with PTB who visited Severance Hospital, which is a 2437-bed tertiary teaching hospital in Seoul, between 2004 and 2020 (31,260 CXR images) were used for model training. Subsequently, data from patients who visited in 2021 (1855 CXR images) were used for testing, while data from those who visited between 2022 and 2023 (3027 CXR images) were used for internal validation. The total data included 4492 patients and 36,142 CXR images (18,557 negative and 17,585 positive). In addition, data from Yongin Severance Hospital, which is a separate 708-bed general hospital located in Yongin, were used for external validation. These included 299 patients and 1978 CXR images, with 863 negative and 1115 positive cases. Figure 1 illustrates the selection process, highlighting the distinct contributions of each institution.

We also used a lung segmentation dataset comprising 704 CXR images, sourced from the National Library of Medicine, National Institutes of Health, United States [16], and the Third People's Hospital of Shenzhen, China [17]. We divided the dataset into 3 subsets, allocating 60% for training, 20% for testing, and 20% for validation.

### Segmentation Model and Cropping Lung Region

**Figure 2:**
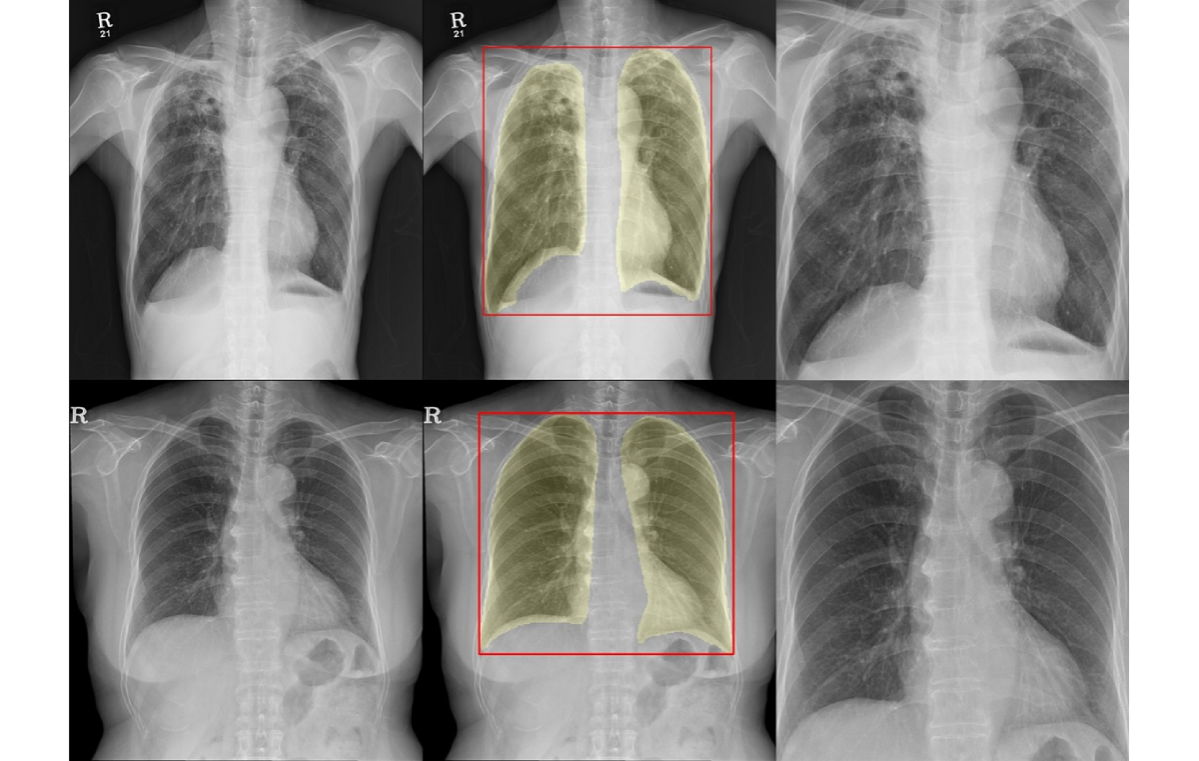
Lung area segmentation and cropping in chest radiographs to enhance tuberculosis infectivity assessment. Left: original chest radiographs; center: segmented and cropped lung area; right: chest radiographs used for analysis.

We developed a lung segmentation model based on TransUNet [18] to focus the analysis on lung parenchyma in the CXR images. Figure 2 shows an example CXR image, where only the lung region is segmented. Following segmentation, only the cropped lung regions were used in the analysis. Further details regarding the segmentation model are provided in Multimedia Appendix 1.

### Convolutional Neural Network and Its Decision-Making Process

We selected the DenseNet121 [19] architecture, which was pretrained using the CheXNet [20] dataset, for developing an infectivity evaluation model. Detailed information regarding the infectivity evaluation model is provided in Multimedia Appendix 2.

In addition, we incorporated gradient-weighted class activation mapping [21]. It visually illustrates the CXR sections on which the neural network focuses during the prediction process through a heat map, highlighting the most influential areas for the predictions. Furthermore, we used local interpretable model-agnostic explanations [22] to segment the CXR images and elucidate the regions that contributed most significantly to its predictions, thereby providing additional insights into its decision-making process.

### Statistical Analysis

This study presents the quantitative data as mean values with SD and categorical data as frequencies and percentages.

In the baseline characteristics, for the analysis of categorical variables, the Fisher exact test was used for “Leukemia” only, whereas the remaining variables were statistically tested using the chi-square test. In addition, “Age” was tested through the Mann-Whitney *U* test. In all statistical tests, a *P* value of <.05 was considered statistically significant. The accuracy, area under the receiver operating characteristic curve (AUROC), area under the precision-recall curve (AUPRC), sensitivity, specificity, positive predictive value (PPV), and negative predictive value (NPV) were used to evaluate the performance of the AI model. We used 1000 bootstrap iterations to compute the 95% CIs for the above metrics.

All analyses were performed using Python (version 3.10.9; Python Software Foundation), TensorFlow-GPU (version 2.10.0; Google), and R (version 4.3.1; R Foundation for Statistical Computing).

### Ethical Consideration

This study adhered to the Declaration of Helsinki guidelines and was approved by the institutional review board of Severance Hospital (4-2023-0679). Owing to its retrospective design, the requirement for individual patient consent was waived by the institutional review board. In addition, we adhered to the Guidelines for Developing and Reporting Machine Learning Predictive Models in Biomedical Research: A Multidisciplinary View [23] throughout our study.

## Results

### Baseline Characteristics

The baseline characteristics of the patients from both hospitals are summarized in Table 1. The proportion of male patients was slightly higher at Severance Hospital (2718/4492, 60.51%) than at Yongin Severance Hospital (178/299, 59.53%), although the difference was not statistically significant (*P*=.91). In addition, the average ages of patients at Severance Hospital and Yongin Severance Hospital were 65 (SD 18.95) years and 66.14 (SD 18.84) years, respectively. This difference in age distribution was not statistically significant (*P*=.22). However, there were significant differences in the prevalence of certain conditions. Chronic obstructive pulmonary disease was more prevalent in the Yongin Severance Hospital group (89/299, 29.77%) than in the Severance Hospital group (914/4492, 20.34%), with a *P* value of <.001.

Similarly, the prevalence of lung cancer and all cancer types was significantly higher in the Severance Hospital group (lung cancer: 255/4492, 5.68%; all cancers: 1200/4492, 26.71%) than in the Yongin Severance Hospital group (lung cancer: 4/299, 1.34%; all cancers: 28/299, 9.36%), both with *P* values of <.001. Other conditions such as drug-induced interstitial lung disease, connective tissue diseases, end-stage renal disease, and diabetes showed no statistically significant differences between the 2 groups (all *P*>.05). In addition, the prevalence of leukemia and pulmonary edema was similar between the 2 groups (leukemia: Severance Hospital 73/4492, 1.63%; Yongin Severance Hospital 6/299, 2%; pulmonary edema: Severance Hospital 78/4492, 1.74%; Yongin Severance Hospital 4/299, 1.34%), with *P* values of .78. Overall, these findings suggest that although there were some statistically significant differences in the prevalence of certain conditions between the patients from the 2 hospitals, other baseline characteristics such as sex, age, and the prevalence of several other conditions were similar.

**Table 1:** Baseline characteristics.

Characteristics	Severance Hospital (n=4492)	Yongin Severance Hospital (n=299)	*P* value
Male, n (%)	2718 (60.51)	178 (59.53)	.91
Age (years), mean (SD)	65 (18.95)	66.14 (18.84)	.22
Chronic obstructive pulmonary disease, n (%)	914 (20.35)	89 (29.77)	<.001
Drug-induced interstitial lung disease, n (%)	109 (2.43)	6 (2.01)	<.001
Lung cancer, n (%)	255 (5.68)	4 (1.34)	<.001
Connective tissue disease, n (%)	170 (3.78)	5 (1.67)	.09
End-stage renal disease, n (%)	560 (12.47)	38 (12.71)	.99
Diabetes, n (%)	1057 (23.53)	88 (29.43)	.09
Leukemia, n (%)	73 (1.63)	6 (2.01)	.78
All cancers, n (%)	1200 (26.71)	28 (9.36)	<.001
Pulmonary edema, n (%)	78 (1.74)	4 (1.34)	.78

#### Infectivity Evaluation

**Figure 3:**
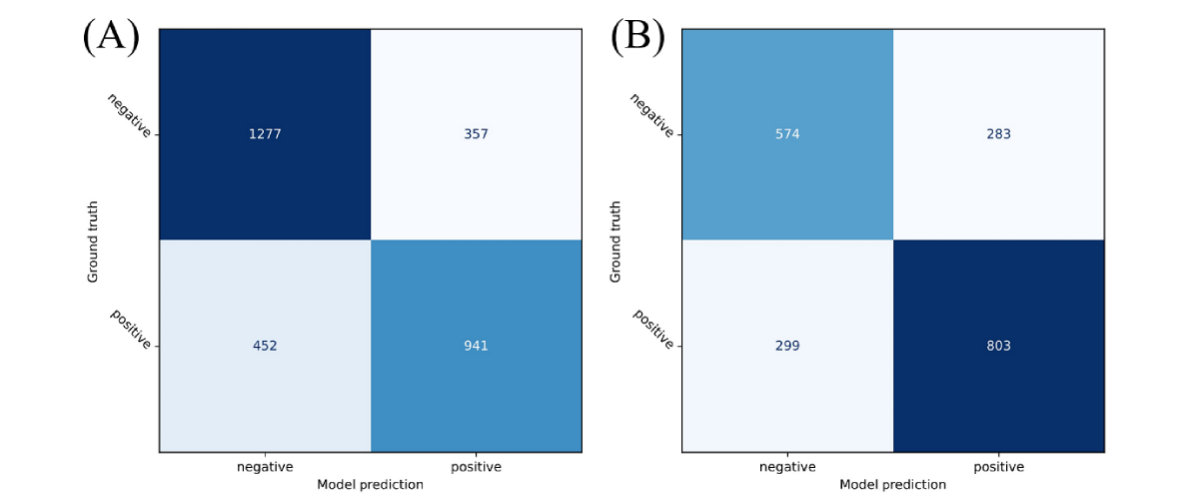
Confusion matrices for (A) internal and (B) external validation of the infectivity evaluation model at a threshold of 0.5.

**Figure 4:**
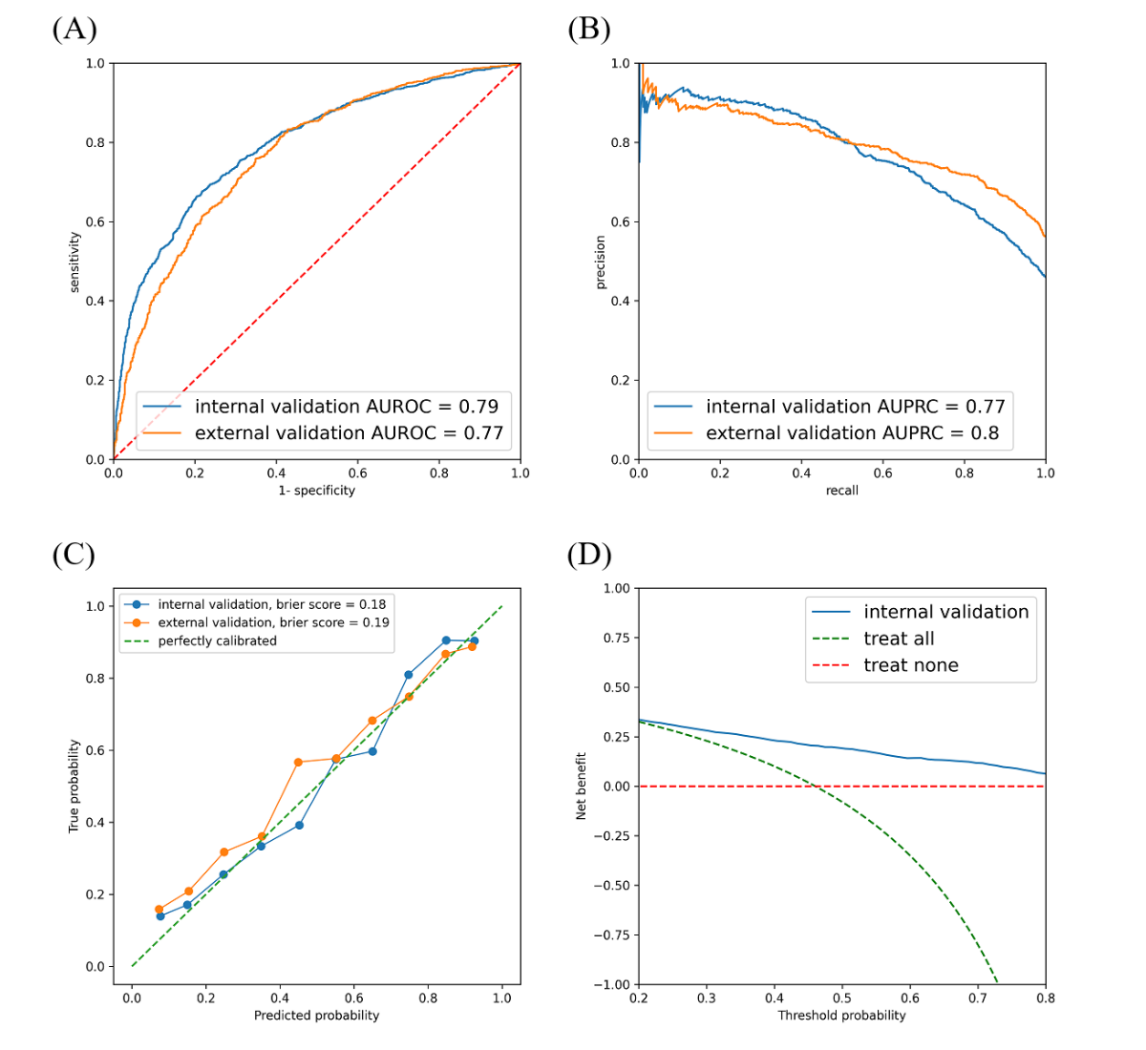
Performance evaluation of the model: internal and external validation results. (A) ROC curve of the model, illustrating the trade-off between sensitivity and specificity, with AUROC values for internal and external validation; (B) PR curve of the model, highlighting precision-recall trade-offs with AUPRC values; (C) calibration plot with Brier scores, showing the agreement between predicted probabilities and observed outcomes; and (D) decision curve analysis, evaluating the net benefit across different threshold probabilities. AUPRC: area under the precision-recall curve; AUROC: area under the receiver operating characteristic curve; PR: precision-Recall; ROC: receiver operating characteristics.

The performance of the infectivity evaluation model was assessed using both internal and external validation sets. The results are summarized in Figure 3 and Table 2. In the internal validation set, the model exhibited an accuracy of 73.27% and a sensitivity of 67.55%, indicating its ability to identify true positive cases correctly, and a specificity of 78.15%, reflecting its effectiveness in identifying true negative cases. Its PPV and NPV were 72.50% and 73.86%, respectively. Its AUROC, which indicates the overall diagnostic ability, was 0.79, whereas its AUPRC was 0.77 (Figure 4A). In the external validation set, the model exhibited an accuracy of 70.29%, a sensitivity of 72.87%, a specificity of 66.98%, and PPV and NPV of 73.94% and 65.75%, respectively. In addition, it achieved AUROC and AUPRC values of 0.77 and 0.8 (Figure 4B), respectively, demonstrating robust performance in differentiating between positive and negative cases on an external validation. We performed calibration plotting to assess how well the predicted probabilities matched the actual outcomes and further evaluate the model. The Brier score, which measures the accuracy of probabilistic predictions, was 0.18 for internal validation and 0.2 for external validation, indicating high levels of calibration and reliability of the probability estimates of the model (Figure 4C). We performed a decision curve analysis to demonstrate clinical use. At nearly all thresholds, the net benefit of using the AI model was higher than that of other strategies. This indicates that the AI model operates effectively across various thresholds, with the highest net benefit observed in the midrange probability thresholds (Figure 4D). The results of comparing the model to other architectures are presented in Multimedia Appendices 3 and 4.

**Table 2:** Infectivity evaluation performance of the artificial intelligence model.

	Accuracy	AUROC^a^	AUPRC^b^	Sensitivity	Specificity	PPV^c^	NPV^d^
Severance Hospital (internal validation), mean (95% CI)	0.73 (0.72-0.75)	0.79 (0.78-0.81)	0.77 (0.75-0.80)	0.68 (0.65-0.70)	0.78 (0.76-0.80)	0.73 (0.70-0.75)	0.74 (0.72-0.76)
Yongin Severance Hospital (external validation), mean (95% CI)	0.7 (0.68-0.73)	0.77 (0.75-0.79)	0.8 (0.77-0.82)	0.73 (0.70-0.76)	0.67 (0.64-0.70)	0.74 (0.72-0.77)	0.66 (0.63-0.69)

^a^AUROC: area under the receiver operating characteristic curve.

^b^AUPRC: area under the precision-recall curve.

^c^PPV: positive predictive value.

^d^NPV: negative predictive value.

#### Visualizing the Decision-Making Process of the AI Model

**Figure 5:**
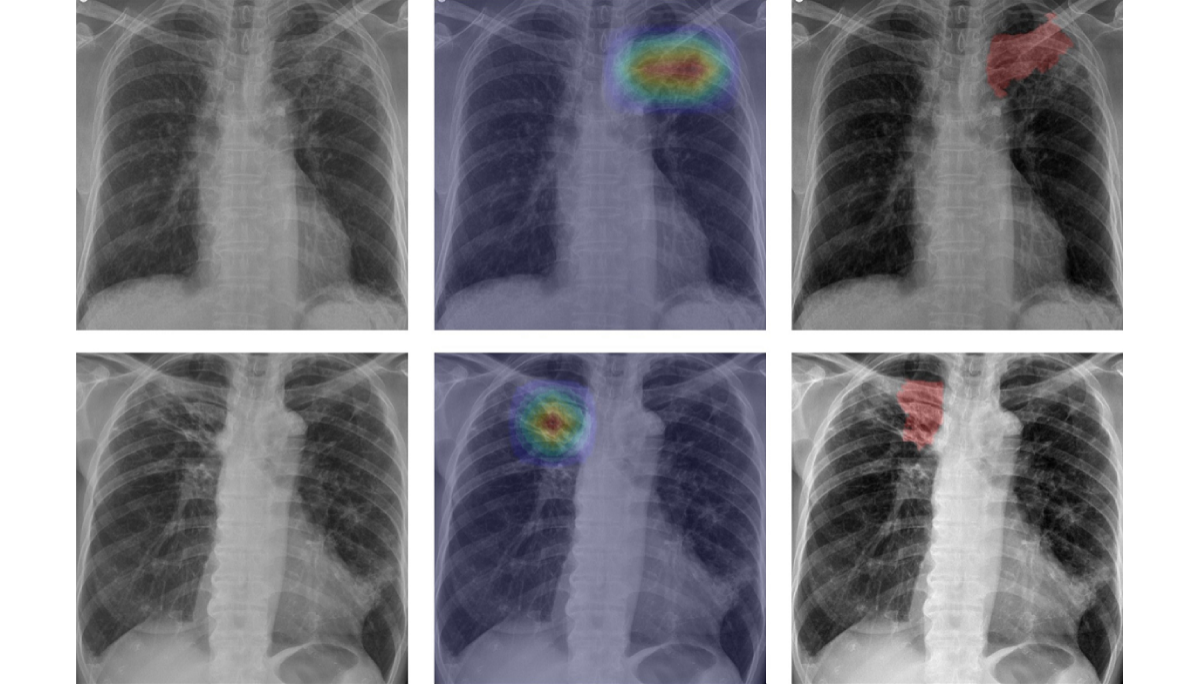
Left: chest radiographs used for the analysis; middle: results of GradCAM; and right: results of LIME. Both the upper and lower panels display chest radiographs that were accurately identified as positive by the model. The analysis in the upper figure focuses on the left lung apex, while the analysis in the lower figure concentrates on the right lung apex, visualizing the areas of frequent tuberculosis occurrences. GradCAM: gradient-weighted class activation mapping; LIME: local interpretable model-agnostic explanations.

In the visualization of the decision-making process of the AI model, it was observed that the model focuses particularly on the left and right lung apex regions. This is consistent with previous findings that the high oxygen concentration and poor lymphatic drainage in the lung apex provide a suitable environment for PTB growth [24]. Thus, it helped in understanding the features that were most indicative of infectivity within the lung regions (Figure 5). These visualization techniques made the model predictions transparent, ensuring that the decision-making process aligned with clinical expectations and knowledge.

#### AI Performance in Evaluating Pulmonary Tuberculosis Infectivity Across Subgroups and Hospitals

Subgroup analyses were conducted among disease groups that showed differences in prevalence in the baseline characteristics, such as chronic obstructive pulmonary disease (COPD) and all cancers between the 2 hospitals, to ascertain whether the model was affected by a particular disease group. The drug-induced interstitial lung disease and lung cancer groups could not be analyzed owing to the presence of only negative cases and the small number of patients in the external validation set. Detailed results for the COPD and all cancers groups between hospitals are presented in Multimedia Appendix 5.

In the analysis between the COPD and non-COPD groups, the AI model exhibited lower sensitivity for both hospitals in the COPD group compared with the non-COPD group. In the comparison between the all cancers and noncancer groups, the all cancers group exhibited higher scores than the noncancer group in several metrics, except for specificity and NPV, at both hospitals.

## Discussion

### Principal Findings

The proposed deep learning model that uses CXR images to evaluate PTB infectivity can facilitate clinical decisions regarding quarantine or discharge as well as assist in evaluating the success of antituberculosis treatments. Furthermore, the ability to differentiate active lesions from healed, old lesions or those being healed can help to avoid unnecessary treatments or evaluations of inactive lesions, which is often an issue in countries with high PTB incidence rates [25,26].

CXR is an important tool for PTB screening, and the World Health Organization recommends its use for this purpose. However, many countries with a high PTB burden have a shortage of trained CXR specialists [27-29]. Therefore, AI-based screening of active PTB could provide these regions with a useful tool to protect public health and enhance PTB control. In addition, according to Kazemzadeh et al [27], deep learning–based, automatic, active PTB screening tools have the potential to offer significant cost savings of 40% to 80% in settings with a prevalence of 1% to 10%.

Furthermore, in countries with moderate to high PTB prevalence, it is common to encounter patients who have been treated or who have spontaneously healed from PTB [28]. Our study focused on distinguishing active, infective PTB, thereby equipping such countries with a more usefully adjusted tool for better PTB control.

Patients who are smear or culture positive for PTB are isolated until their sputum microbiological tests return negative, which can take several weeks for confirmation [30]. Our infectivity-targeted model offers the potential for immediate assessment, which could facilitate quicker decisions regarding discharge from the hospital or return to the workplace, potentially benefiting the socioeconomic situation of the region.

The proposed model exhibited consistent and reliable performance in both internal and external validations, highlighting its clinical applicability for determining PTB infectivity. By achieving a crucial balance between sensitivity and specificity, it effectively differentiated infective and noninfective cases, which holds significant clinical importance. Moreover, the integration of visualization techniques improved its transparency by elucidating the specific areas that influenced its decisions. Thus, the proposed model can aid in PTB management and assessment by leveraging broadly accessible CXR images.

Traditionally used smear tests have low sensitivity, which can lead to missed diagnoses, especially in cases of early infections with low bacterial loads. Although culture tests are considered standard, they can be slow, requiring several weeks to produce results. Thus, diagnostic decision-making must be improved through more efficient methods. Table 3 lists the benefits and limitations of the smear, culture, and CXR tests, providing insights into their performance, speed, and overall efficacy for diagnosing PTB. Evidently, the proposed CXR test can produce diagnosis results within several seconds, enabling rapid decision-making for quarantine. Moreover, it is noninvasive, cost-effective, and widely accessible and features minimal patient discomfort. In addition, advances in AI have enabled automated analyses, which reduce clinician workloads and improve diagnostic accuracy.

**Table 3:** Comparison of smear, culture, and chest radiography tests.

	Culture [31]	Smear [32]	Chest radiography (ours)
Sensitivity	80.8%	46.2%	67.6%
Specificity	99.0%	99.3%	78.2%
Result time	Within weeks	Within hours	Within seconds
Advantages	High sensitivity	High specificity	Automated, rapid test results
Limitations	Long test results, contamination	Low sensitivity, low bacterial load	Low specificity

CT has also been used to differentiate PTB cases. Gao et al [33] developed and evaluated TBINet, a deep learning model that uses CT images to determine the infectivity of patients with PTB. It achieved AUROC scores of 0.82 and 0.75 for the validation and external validation sets, respectively, thereby outperforming traditional deep learning methods. However, the limitations of CT include high costs, limited facilities, and radiation exposure [34]. Our approach overcomes these limitations by using the more accessible and safer CXR, which is performed similarly to CT-based models. Choi et al [35] and Lee et al [28] have demonstrated the potential of CXR to effectively distinguish between patients with active PTB and healthy patients. Their findings, highlighted by AUROC scores ranging from 0.83 to 0.98, underscored the high accuracy of CXR-based techniques for identifying PTB. Existing studies have primarily focused on using AI for the diagnosis of PTB using CXR [36,37], but the potential of AI to monitor the progress of patients undergoing treatment has been relatively less explored. Unlike the studies mentioned above, which compared healthy patients against patients with PTB, our study differentiated between infectious and noninfectious cases to assess infectivity. Our study proposes the potential use of AI models to analyze CXR images of patients undergoing treatment, enabling the monitoring of treatment progress. In addition, our model can aid in determining the appropriate timing for lifting unquarantined measures. By assessing whether a patient remains infectious, health care providers can make more informed decisions about when it is safe to release patients from quarantine. This not only helps prevent the disease transmission but also reduces the psychological and social burden on patients who may otherwise face longer periods of quarantine than necessary.

Our model facilitates faster decision-making by capitalizing on the quick and accessible nature of CXR, which allows imaging within minutes, and the capability of the proposed model to evaluate CXR images in less than a second. This rapid turnaround time can lead to faster patient discharge or quarantine decisions, emphasizing its efficiency and effectiveness in clinical settings. Given the distinct challenges addressed in this study, comparing our AUROC scores directly with those of other studies may not fully capture its unique value and contributions, which lie in its innovative focus on distinguishing between PTB infectivity stages, rather than merely identifying active cases.

In addition, this study focused on the lung regions in CXR images and used a refined method for segmentation and cropping to accurately target the areas of interest. Thus, it not only improves the diagnostic accuracy by providing a clearer view of the lung regions but also significantly minimizes the impact of irrelevant factors on the decision-making process of the AI model. Building on the groundwork laid by Zaidi et al [38], who showed that lung segmentation can enhance the classification results of CXR analysis by reducing noise and improving efficiency, this study achieved further performance enhancements by exclusively concentrating on segmented and cropped lung areas. Thus, it demonstrated the substantial potential of focused image analysis for improving the accuracy and reliability of AI-driven diagnostics using medical images.

Moreover, our study used large-scale data that were collected over a broad time span, from 2004 to 2023, which was advantageous for analyzing temporal changes and trends in PTB diagnoses and treatments. In addition, data from almost 2 decades allowed us to draw more accurate and reliable conclusions, providing a robust foundation for the AI model to learn and predict with precision. Thus, it offers significant advancements in AI applications to medical imaging, particularly for PTB diagnosis and management, and provides a reliable and clinically applicable tool.

Furthermore, we established a 1-month gray zone following a change in the sputum test results of the patient for labeling purposes. This was necessary to enhance the certainty of the infection status of the patient after the sputum test result became negative because a negative sputum test result does not immediately indicate that the patient is no longer infected. We excluded CXR images obtained during this period to enhance the accuracy for determining when the likelihood of the contagion decreased to aid in patient treatment and management. This 1-month period was selected based on its relevance compared with 2, 4, 6, and 8 weeks. However, it is important to recognize that using this gray zone could potentially reduce the discriminative power of the model owing to the overlap of continuous and minor changes in images throughout the treatment process. Although extending the gray zone could improve the discrimination power, we elected not to extend it beyond 1 month, which is the minimum interval for follow-ups to assess the infectivity reduction during treatment.

The results of the decision curve analysis demonstrated that the AI model maintained the highest net benefit across most threshold probabilities. This indicates that the AI model is effective in accurately identifying patients with infectious PTB while reducing unnecessary treatments. These findings suggest that the model can significantly contribute to public health efforts in reducing the spread of PTB.

According to the subgroup analysis comparing the model evaluation for the COPD and non-COPD groups, the sensitivity of the model was lower in patients with COPD than in the non-COPD group. This may be because COPD has different pathological features in CXR, including various degrees of inflammation of the lungs, hyperinflation and emphysema, and consolidations and bronchiolitis, which are causes of acute exacerbation episodes [39]. When these features are overlaid with typical CXR findings of PTB infectivity, it may be difficult for AI models to evaluate PTB infectivity, which means that the presence of COPD may disrupt markers of PTB infectivity [40,41], leading to reduced sensitivity.

In the comparison of the all cancers and noncancer groups, we found that the model’s evaluation for the noncancer group was lower than that for all cancers for most metrics, except for specificity and NPV. This is because most patients with cancer had been treated with, or were receiving, chemotherapeutic agents. These treatments may impair their cellular and humoral immune systems. Moreover, lung cancer could adversely affect the immune response to PTB [42,43], potentially exacerbating PTB [44], which leads to pronounced changes on CXR [45]. Thus, the AI model may have been better equipped to evaluate signs of infectivity in the CXR images of these patients. In addition, active tuberculosis can increase the risk of cancer, and a cancer diagnosis can also heighten the risk of tuberculosis owing to a mutual interaction [46]. Consequently, the higher sensitivity of AI in detecting PTB in patients with cancer could assist in the screening of tuberculosis in these patients.

### Limitations

**Figure 6:**
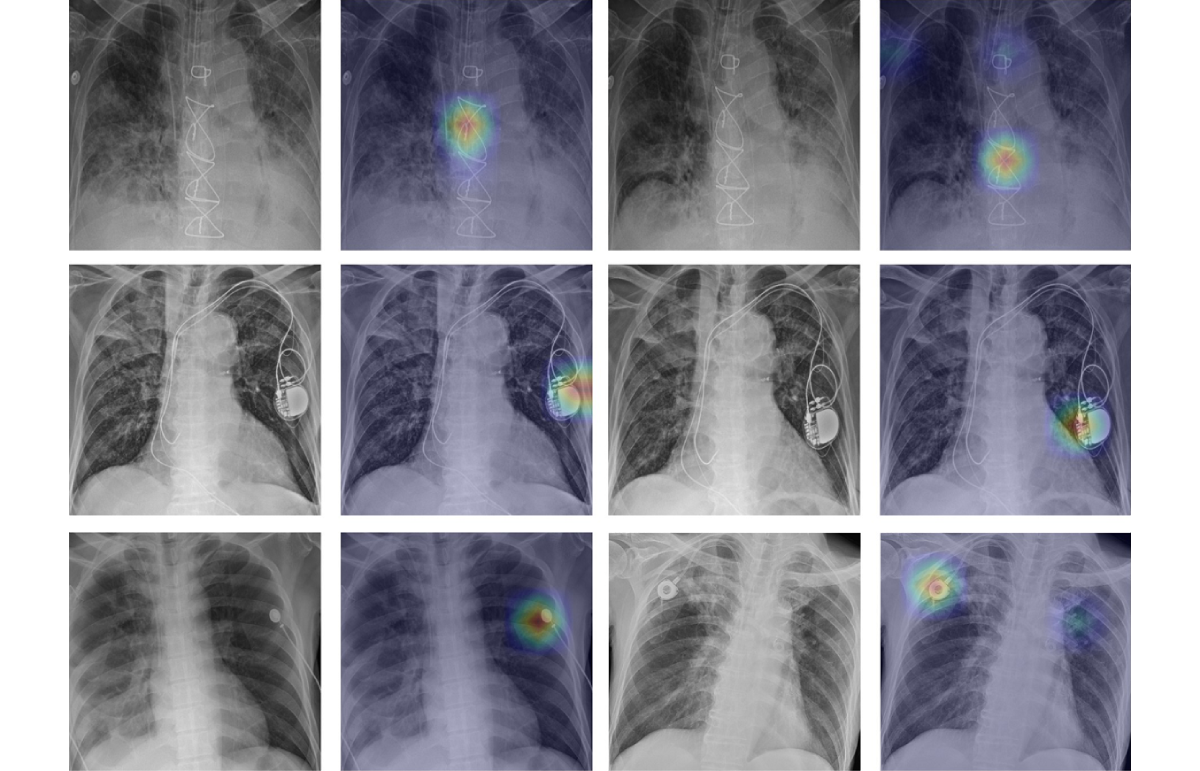
The upper, middle, and lower cases are all positive. The AI evaluated the upper case as positive due to the sternotomy suture material, the middle case due to the pacemaker region, the lower left case due to the EKG leads, and the lower right case due to the chemoport catheter. AI: artificial intelligence; EKG: electrocardiogram.

Although the proposed AI model exhibited notable performance, it has certain limitations. First, although interpretation techniques aid in understanding the decisions of the AI model and provide valuable insights, their interpretations can be complex. While these methods effectively highlight regions significant to the AI decision-making process, such as the right and left upper lobes commonly affected by PTB, they sometimes focus on areas that pose challenges for straightforward clinical correlation. This indicates that although the areas of focus of the proposed AI model were aligned with known PTB-affected regions, these techniques underscore the need for further research to elucidate specific infectivity biomarkers in images. In addition, in some CXR images, the model focused on regions with foreign objects, such as sternotomy suture material, Electrocardiogram leads, and pacemakers, interpreting them as positive cases. These objects may act as confounders. Figure 6 depicts some of these CXRs.

Second, although a 1-month gray period was used to evaluate changes in the infection status, it remains uncertain whether this timeframe is universally applicable to all patients or offers adequate flexibility for diverse scenarios.

Third, although this study emphasized the PTB diagnosis accuracy of the model, acknowledging the inherent constraints of retrospective research, it did not assess the long-term effects on patient outcomes following the application of the AI tool, indicating the necessity for future prospective studies.

Fourth, it included only patients who were successfully treated through a 6-month short-course treatment regimen; this methodology omits a range of treatment outcomes, including delayed response, treatment failure, and multidrug-resistant tuberculosis, and may therefore fail to encompass all phases of the tuberculosis treatment continuum.

Finally, the model may have a bias toward Koreans as the study patients were only Korean. Further experiments with multicountry data, including different ethnicities, are required to address this.

### Conclusion

In conclusion, this study represents a step forward in tackling the challenges associated with PTB diagnosis by introducing an AI tool that uses CXR images to assess PTB infectivity. The proposed AI model and interpretation techniques can evaluate PTB infectivity and elucidate its decision-making process, thereby aiding in decisions regarding patient discharge or quarantine.

Therefore, the proposed approach aims to minimize the spread of PTB by identifying individuals who pose a risk of infection. Importantly, it offers a more accurate and expedited screening process than conventional smear tests and provides quicker results than culture methods, with the potential to significantly enhance the efficiency and effectiveness of PTB diagnosis and management.

## Appendix

Multimedia Appendix 1 Detailed optimization strategies and hyperparameters in the TransUNet training process.

Multimedia Appendix 2 Detailed optimization strategies and hyperparameters in the DenseNet121 training process.

Multimedia Appendix 3 Results of comparative experiments with different artificial intelligence (AI) model architectures.

Multimedia Appendix 4 Results from each artificial intelligence (AI) model.

Multimedia Appendix 5 Performance comparison for COPD, non-COPD, all cancer, and noncancer groups across internal and external validations. COPD: chronic obstructive pulmonary disease.

## Abbreviations

AI: artificial intelligence
AUPRC: area under the precision-recall curve
AUROC: area under the receiver operating characteristic curve
COPD: chronic obstructive pulmonary disease
CT: computed tomography
CXR: chest radiography
NPV: negative predictive value
PPV: positive predictive value
PTB: pulmonary tuberculosis

## Data Availability

The datasets generated during and/or analyzed during this study are available from the corresponding author on reasonable request. The developed infectivity evaluation and segmentation models are available on our GitHub repository [47].
