# Supplementary material for: Development and Validation of Deep Learning–Based Infectivity Prediction in Pulmonary Tuberculosis Through Chest Radiography: Retrospective Study
Source: J Med Internet Res. 2024 Nov 7;26:e58413. doi: 10.2196/58413 (PMC11582483; doi:10.2196/58413)
Supplement: Multimedia Appendix 1 [file jmir_v26i1e58413_app1.docx]

**Multimedia Appendix 1, Detailed Optimization Strategies and Hyperparameters in the TransUNet Training Process.**

TransUNet exhibits superior accuracy over traditional convolutional neural networks (CNNs), using Transformer components to enhance global information processing. This synergy with the structure of U-Net optimizes feature learning for lung segmentation. The Dice coefficient of the model was 0.97.

Data augmentation was applied by flipping the CXRs horizontally and vertically, and by rotating them in increments of 1 degree from 1 to 15 degrees.

Hyperparameters used in the training process were as follows: all CXRs were resized to (512,512) pixels, the grayscale CXR pixel values were normalized to a range of 0 to 1 by dividing by 255. A batch size of 8 was used. The loss function used was dice loss, the optimizer was AdamW with weight decay 1e-6. The learning rate was set at 1e-4, with a learning rate reduction technique applied to gradually decrease it to 1e-6, and early stopping with a patience of 5 based on validation loss was applied to select the model with the lowest validation loss. Epoch 27 had the smallest validation loss of 0.03, and the model from that epoch was used finally.

The hyper-parameters for the model structure are as follows:

The number of filters : [64, 128, 256, 512]

The number of stacks in down-sampling : 2

The number of stacks in up-sampling : 2

embedding dimension :768

The number of MLP units :2048

The number of attention heads :4

The number of transformer blocks : 4

activation function : relu

MLP activation function : gelu

Output activation function : sigmoid

Up-sampling method : bilinear
