# Supplementary material for: Development and Validation of Deep Learning–Based Infectivity Prediction in Pulmonary Tuberculosis Through Chest Radiography: Retrospective Study
Source: J Med Internet Res. 2024 Nov 7;26:e58413. doi: 10.2196/58413 (PMC11582483; doi:10.2196/58413)

**Multimedia Appendix 2, Detailed Optimization Strategies and Hyperparameters in the DenseNet121 Training Process.**

We used a fine-tuning technique for infectivity assessment. DenseNet121 is a sophisticated AI model that is designed for intricate image analysis tasks and distinguished by its multiple interconnected layers, each of which receives from all preceding layers. This unique structure ensures exceptionally efficient and accurate pattern recognition from images, making it particularly suitable for medical applications in CXR analysis.

Data augmentation was applied by flipping the CXRs horizontally and vertically, and by rotating them in increments of 1 degree from 1 to 15 degrees.

We have trained the model using Grid Search for optimization. Hyperparameters used in the training process were as follows: all CXRs were resized to (224,224) pixels. the grayscale CXR pixel values were normalized to a range of 0 to 1 by dividing by 255. A batch size of 32 was used. The loss function used was binary crossentropy, the optimizer was AdamW with weight decay 1e-6. The learning rate of the pre-trained DenseNet121 was set to 1e-6 for fine-tuning and all layers were trainable, while for the rest it was set at 1e-4, with a learning rate reduction technique applied to gradually decrease it to 1e-6, and early stopping with a patience of 5 based on validation loss was applied to select the model with the lowest validation loss. Epoch 19 had the smallest validation loss of 0.13, and the model from that epoch was used finally.

The hyper-parameters for the model structure illustrated as follows: The dropout rate was 0.2, and the activation function for the Fully Connected Layer used leaky_relu.


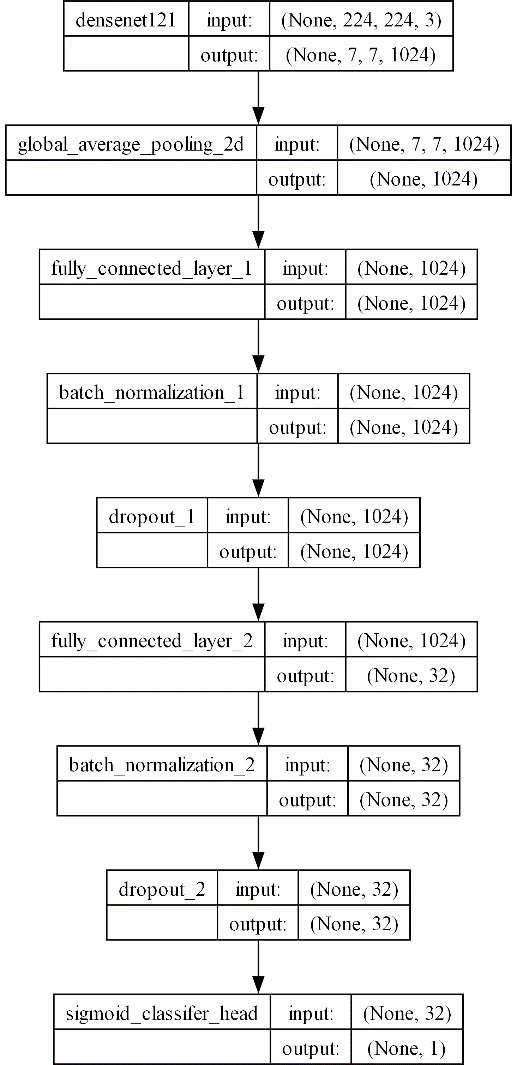

Supplement: Multimedia Appendix 2 [file jmir_v26i1e58413_app2.docx]
