# Supplementary material for: Development and Validation of Deep Learning–Based Infectivity Prediction in Pulmonary Tuberculosis Through Chest Radiography: Retrospective Study
Source: J Med Internet Res. 2024 Nov 7;26:e58413. doi: 10.2196/58413 (PMC11582483; doi:10.2196/58413)
Supplement: Multimedia Appendix 3 [file jmir_v26i1e58413_app3.docx]

**Multimedia Appendix 3, Results of comparative experiments with different AI model** **architectures.**

|  | Accuracy | AUROC | AUPRC | Sensitivity | Specificity | PPV | NPV |
| --- | --- | --- | --- | --- | --- | --- | --- |
| Internal validation |  | | | | | | |
| Vit B16 | 0.6501 | 0.7713 | 0.7439 | 0.8557 | 0.4749 | 0.5815 | 0.4749 |
| EfficientNetV2L | 0.6720 | 0.7399 | 0.5922 | 0.9229 | 0.2637 | 0.6171 | 0.7267 |
| ResNet152V2 | 0.6350 | 0.7267 | 0.6874 | 0.8011 | 0.4933 | 0.5741 | 0.7442 |
| DenseNet201 | 0.6683 | 0.7063 | 0.6714 | 0.7976 | 0.4541 | 0.5547 | 0.7246 |
| DenseNet121 (Pretrained) | 0.7327 | 0.7917 | 0.7716 | 0.6755 | 0.7815 | 0.7250 | 0.7386 |
| External validation |  | | | | | | |
| Vit B16 | 0.6462 | 0.7311 | 0.7406 | 0.9428 | 0.2649 | 0.6225 | 0.7828 |
| EfficientNetV2L | 0.6345 | 0.6932 | 0.7226 | 0.9229 | 0.2637 | 0.6171 | 0.7267 |
| ResNet152V2 | 0.6197 | 0.7011 | 0.7181 | 0.9528 | 0.1914 | 0.6024 | 0.7593 |
| DenseNet201 | 0.6136 | 0.6804 | 0.7145 | 0.9247 | 0.2135 | 0.6019 | 0.6880 |
| DenseNet121 (Pretrained) | 0.7029 | 0.7686 | 0.7970 | 0.7287 | 0.6698 | 0.7394 | 0.6575 |

Shading indicates best performance.
