# Supplementary material for: Development and Validation of Deep Learning–Based Infectivity Prediction in Pulmonary Tuberculosis Through Chest Radiography: Retrospective Study
Source: J Med Internet Res. 2024 Nov 7;26:e58413. doi: 10.2196/58413 (PMC11582483; doi:10.2196/58413)

**Multimedia Appendix** **4, Results from each AI model.**

Confusion matrix and ROC, PR Curves for Vit B16


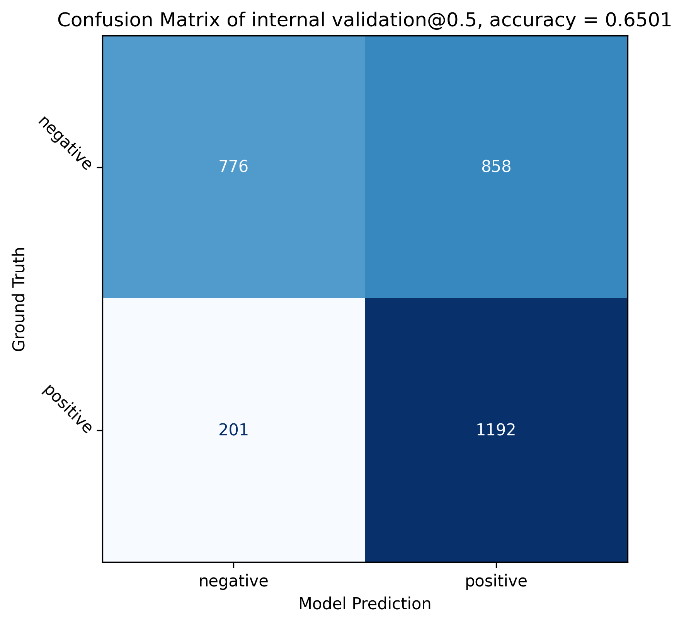

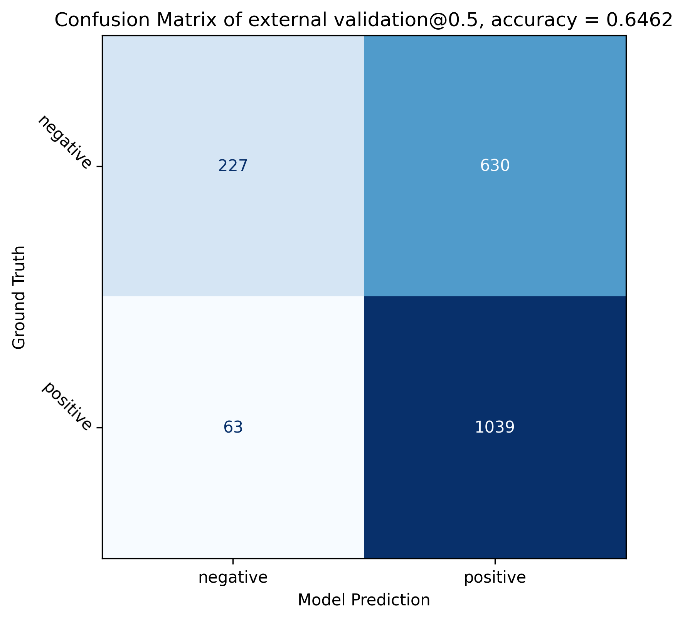


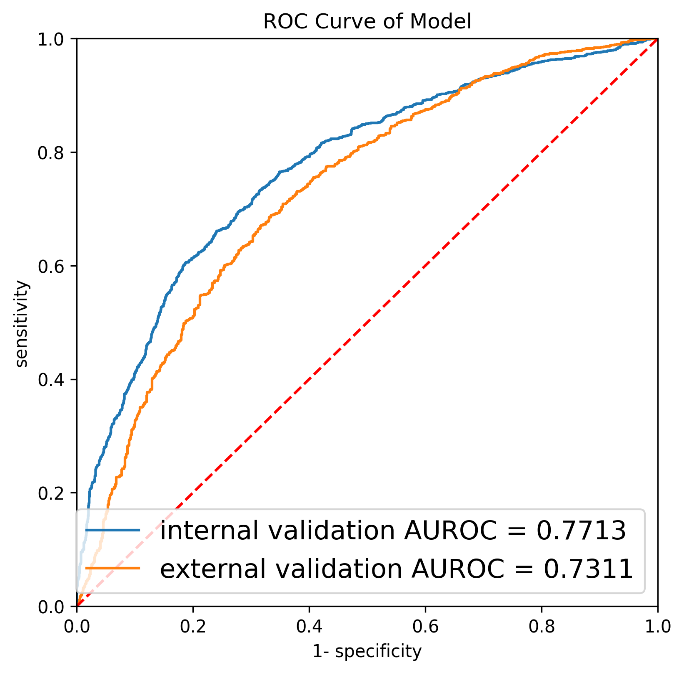

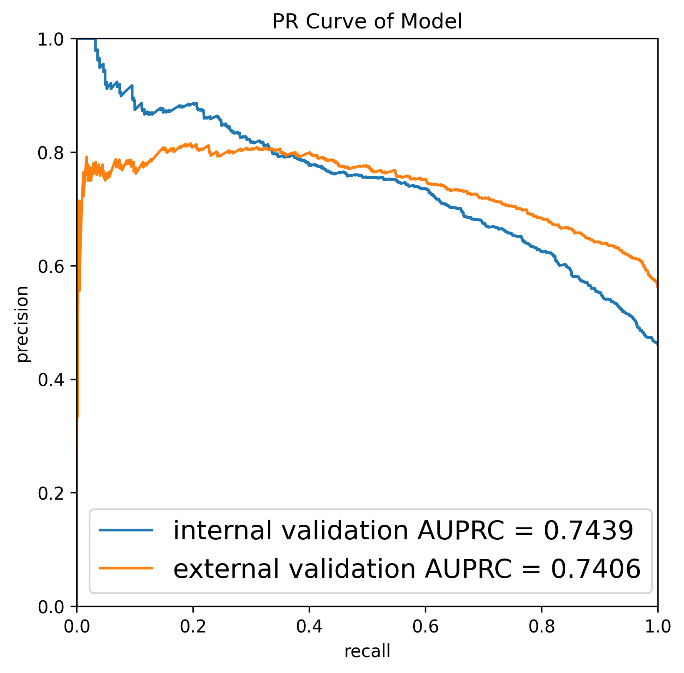


Confusion matrix and ROC, PR Curves for EfficientNetV2L


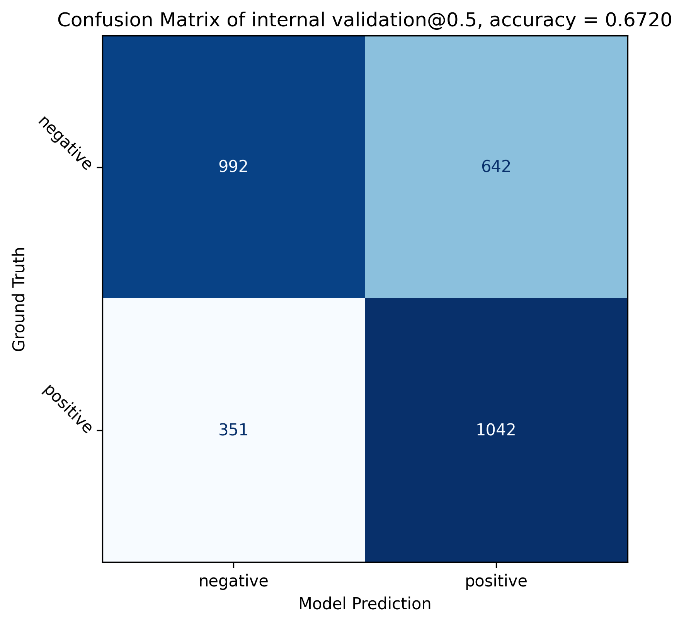

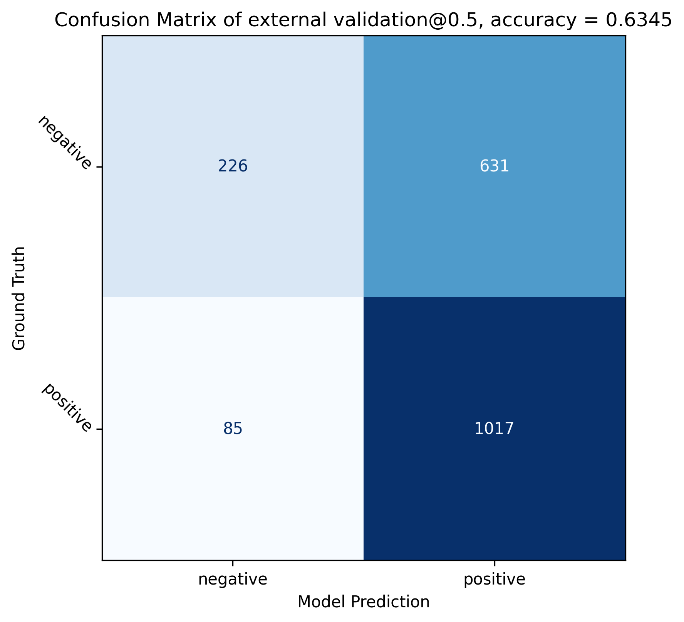

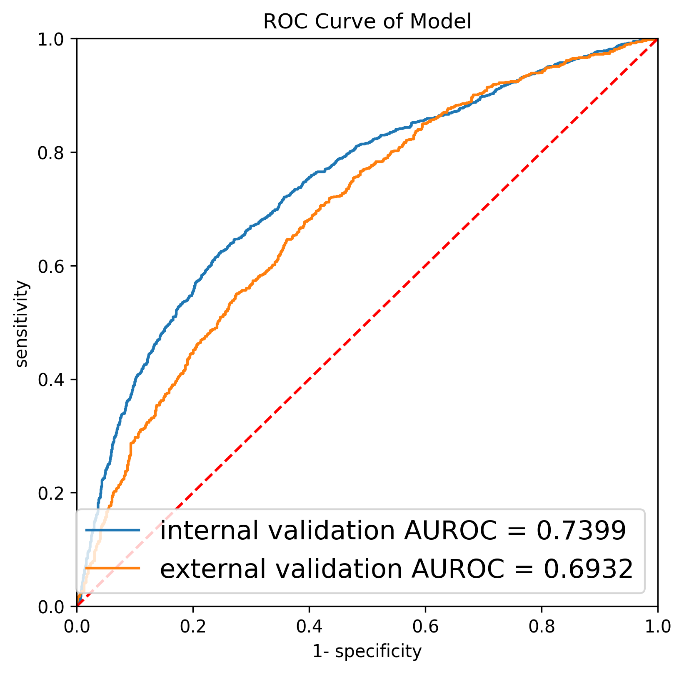

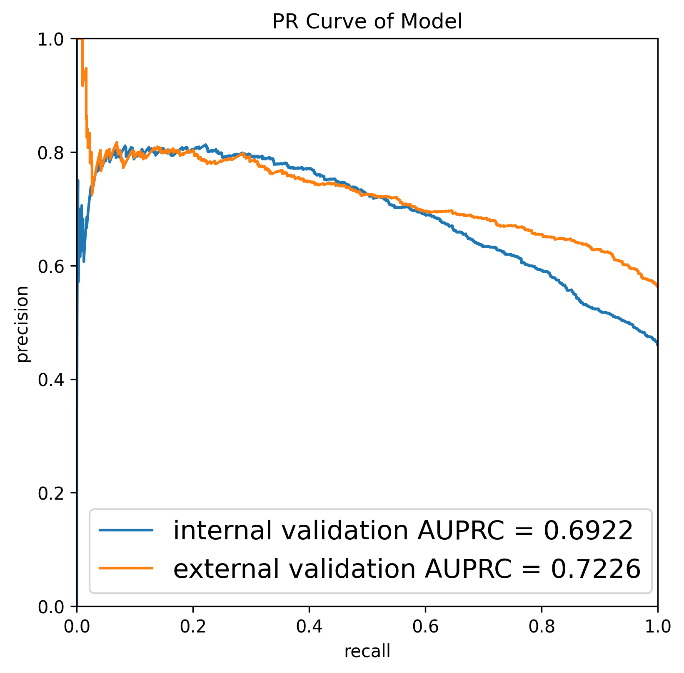


Confusion matrix and ROC, PR Curves for ResNet152V2


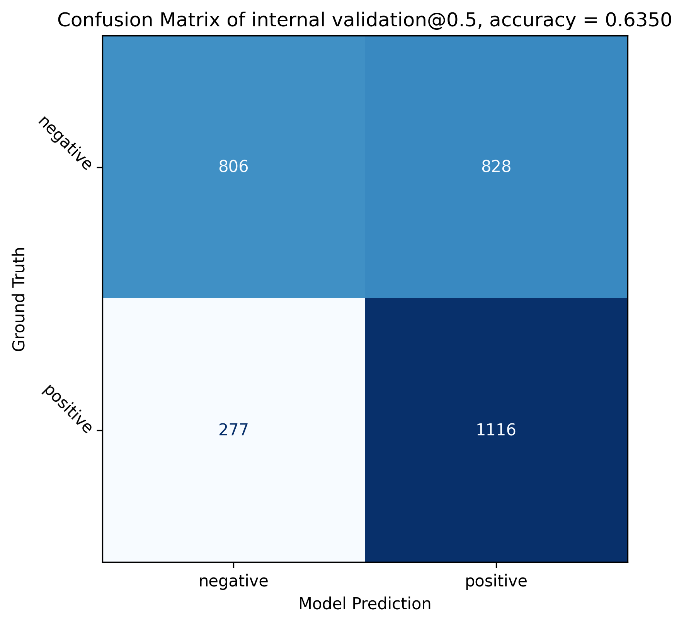

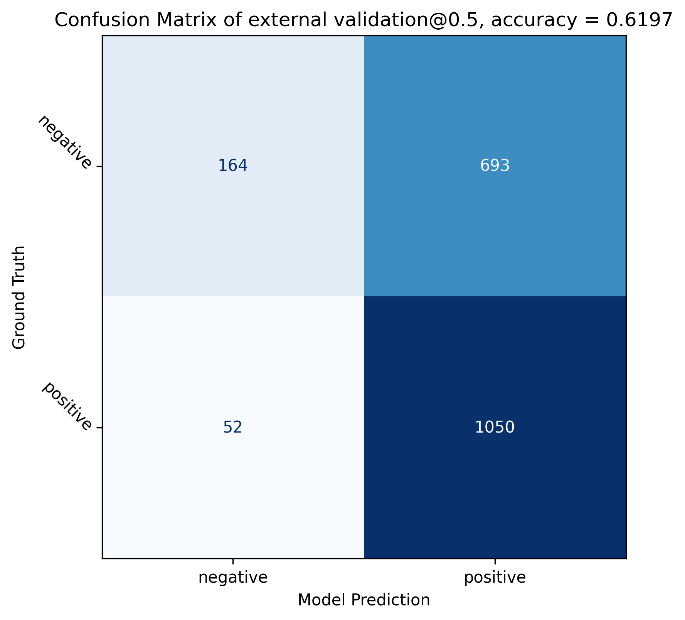

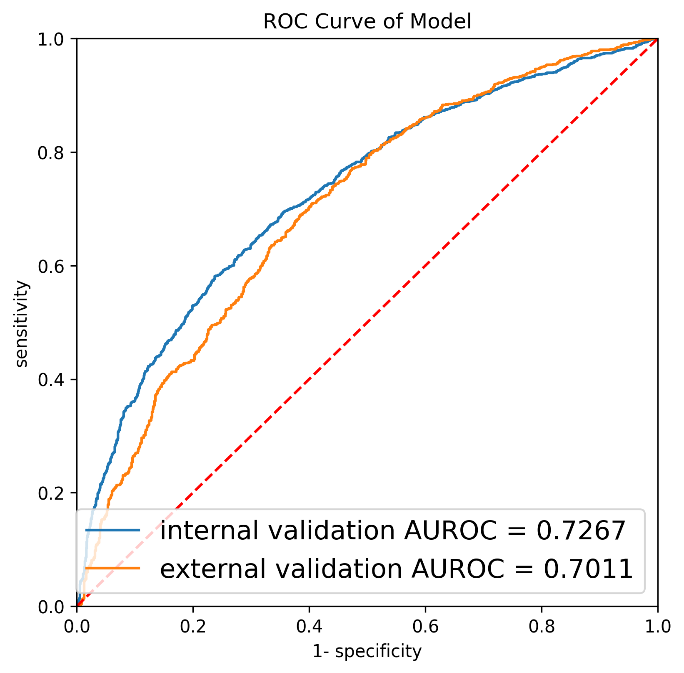

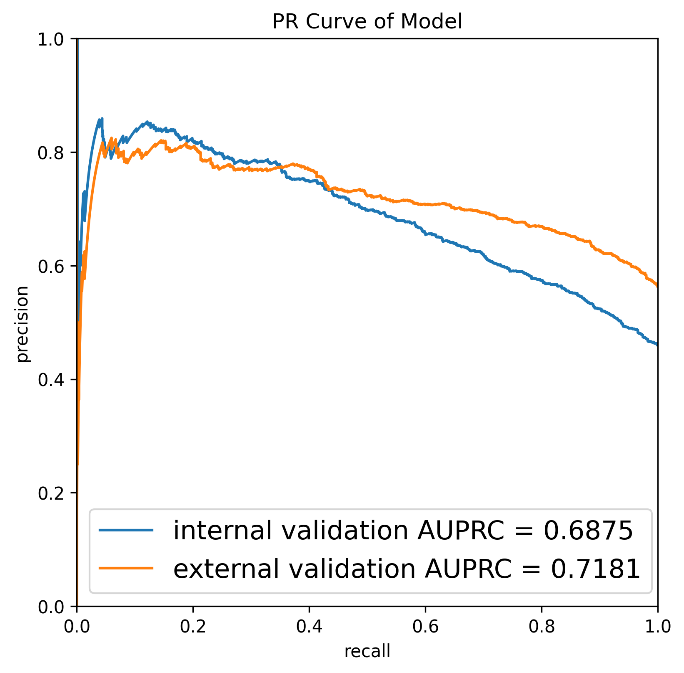


Confusion Matrix and ROC, PRC Curves for DenseNet201


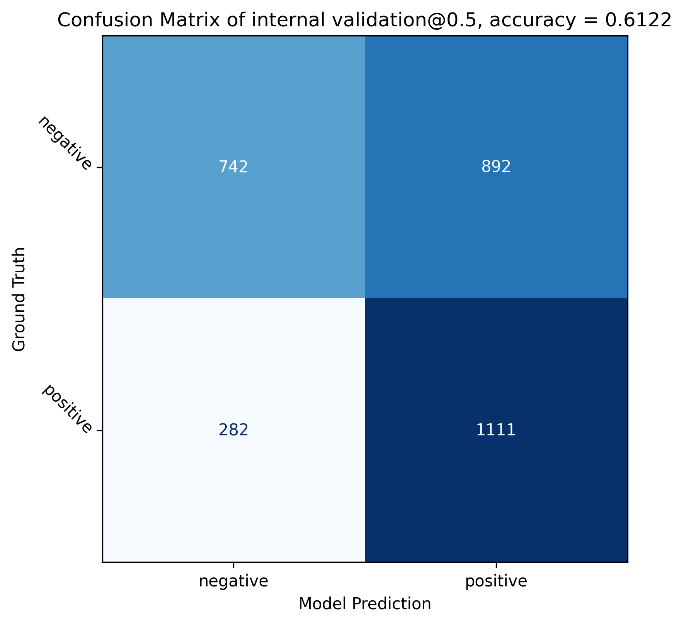

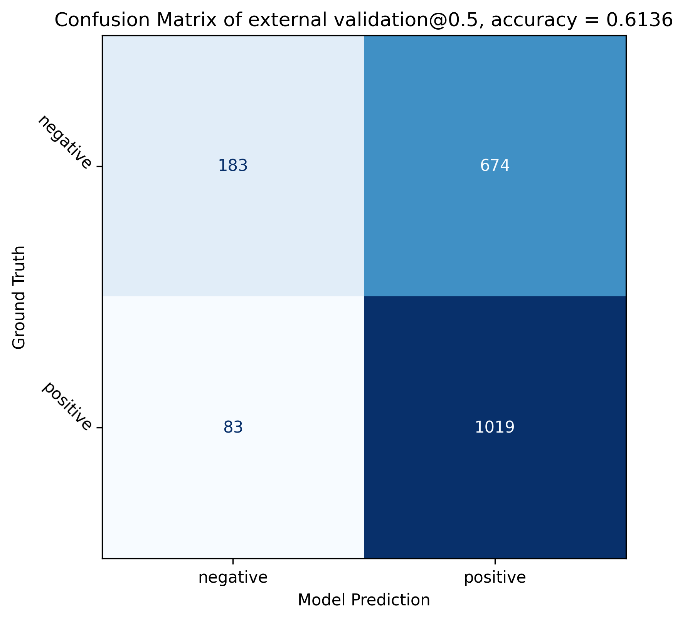

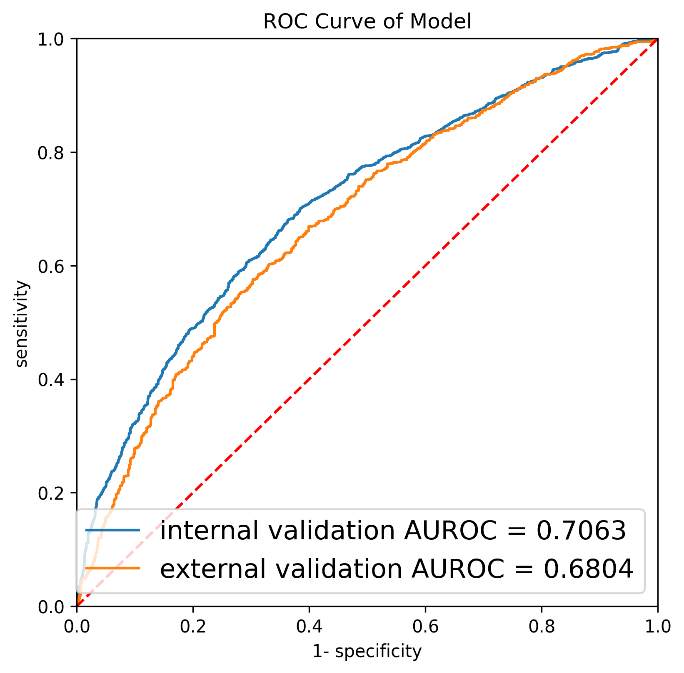

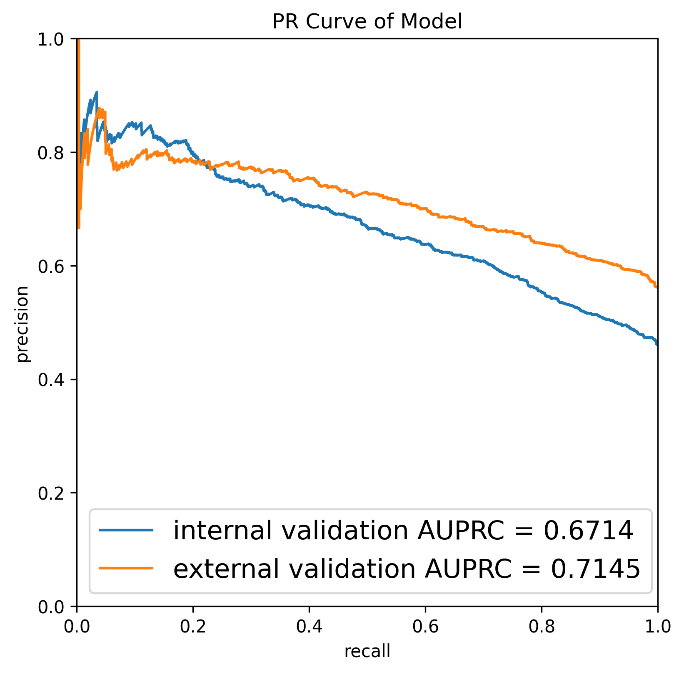

Supplement: Multimedia Appendix 4 [file jmir_v26i1e58413_app4.docx]
