# Supplementary material for: Development and Validation of Deep Learning–Based Infectivity Prediction in Pulmonary Tuberculosis Through Chest Radiography: Retrospective Study
Source: J Med Internet Res. 2024 Nov 7;26:e58413. doi: 10.2196/58413 (PMC11582483; doi:10.2196/58413)
Supplement: Multimedia Appendix 5 [file jmir_v26i1e58413_app5.docx]

**Multimedia Appendix** **5, Performance comparison for COPD, Non-COPD, All Cancer, and Non-Cancer groups across internal and external validation.**

|  | Accuracy | AUROC | AUPRC | Sensitivity | Specificity | PPV | NPV |
| --- | --- | --- | --- | --- | --- | --- | --- |
| internal validation |  |  |  |  |  |  |  |
| COPD | 0.7209  (0.67, 0.77) | 0.7684  (0.72, 0.82) | 0.7866  (0.72, 0.84) | 0.5833  (0.53, 0.63) | 0.8471  (0.79, 0.90) | 0.7778  (0.70, 0.85) | 0.6890  (0.62, 0.75) |
| Non-COPD | 0.7279  (0.71, 0.74) | 0.7904  (0.77, 0.81) | 0.7758  (0.76, 0.80) | 0.6667  (0.64, 0.71) | 0.7783  (0.76, 0.80) | 0.7121  (0.68, 0.74) | 0.7395  (0.72, 0.76) |
| All Cancers | 0.7952  (0.77, 0.82) | 0.8446  (0.82, 0.88) | 0.8294  (0.80, 0.88) | 0.7938  (0.75, 0.83) | 0.7968  (0.76, 0.84) | 0.8151  (0.78, 0.85) | 0.7740  (0.73, 0.82) |
| Non-Cancers | 0.7104  (0.69, 0.73) | 0.7673  (0.75, 0.79) | 0.7389  (0.71, 0.77) | 0.6241  (0.59, 0.65) | 0.7770  (0.75, 0.80) | 0.6832  (0.65, 0.72) | 0.7284  (0.70, 0.75) |
| external validation |  |  |  |  |  |  |  |
| COPD | 0.6178  (0.55, 0.69) | 0.6916  (0.63, 0.79) | 0.7457  (0.67, 0.84) | 0.5977  (0.49, 0.70) | 0.6429  (0.53, 0.76) | 0.6753  (0.57, 0.78) | 0.5625  (0.45, 0.67) |
| Non-COPD | 0.7129  (0.69, 0.73) | 0.7792  (0.76, 0.80) | 0.8063  (0.78, 0.83) | 0.7396  (0.71, 0.77) | 0.6782  (0.65, 0.71) | 0.7493  (0.72, 0.78) | 0.6671  (0.63, 0.70) |
| All Cancers | 0.7727  (0.70, 0.83) | 0.9018  (0.85, 0.94) | 0.9536  (0.93, 0.97) | 0.7857  (0.71, 0.86) | 0.7500  (0.65, 0.86) | 0.8462  (0.78, 0.91) | 0.6667  (0.55, 0.77) |
| Non-Cancers | 0.7021  (0.68, 0.72) | 0.7675  (0.75, 0.79) | 0.7958  (0.77, 0.82) | 0.7279  (0.70, 0.76) | 0.6690  (0.64, 0.70) | 0.7381  (0.71, 0.76) | 0.6574  (0.62, 0.69) |

Values in parentheses represent 95% confidence intervals.
